# Cell Membrane-Coated Biomimetic Nanoparticles in Cancer Treatment

**DOI:** 10.3390/pharmaceutics16040531

**Published:** 2024-04-12

**Authors:** Shu Zhang, Xiaojuan Zhang, Huan Gao, Xiaoqin Zhang, Lidan Sun, Yueyan Huang, Jie Zhang, Baoyue Ding

**Affiliations:** 1 School of Pharmaceutical Sciences, Zhejiang Chinese Medical University, Hangzhou 214122, China; zshuee@163.com; 2 Jiaxing Key Laboratory for Photonanomedicine and Experimental Therapeutics, Department of Pharmaceutics, College of Medicine, Jiaxing University, Jiaxing 314001, China; xjzhang@zjxu.edu.cn (X.Z.); gaohuanbxg@163.com (H.G.); qin@zjxu.edu.cn (X.Z.); slidan89@zjxu.edu.cn (L.S.); hyylinda@163.com (Y.H.)

**Keywords:** cell membrane-coated nanoparticles, drug delivery, biomimetic, tumor targeting

## Abstract

Nanoparticle-based drug delivery systems hold promise for cancer treatment by enhancing the solubility and stability of anti-tumor drugs. Nonetheless, the challenges of inadequate targeting and limited biocompatibility persist. In recent years, cell membrane nano-biomimetic drug delivery systems have emerged as a focal point of research and development, due to their exceptional traits, including precise targeting, low toxicity, and good biocompatibility. This review outlines the categorization and advantages of cell membrane bionic nano-delivery systems, provides an introduction to preparation methods, and assesses their applications in cancer treatment, including chemotherapy, gene therapy, immunotherapy, photodynamic therapy, photothermal therapy, and combination therapy. Notably, the review delves into the challenges in the application of various cell membrane bionic nano-delivery systems and identifies opportunities for future advancement. Embracing cell membrane-coated biomimetic nanoparticles presents a novel and unparalleled avenue for personalized tumor therapy.

## 1. Introduction

Cancer, a grave and increasingly prevalent disease, is anticipated to account for approximately 1.96 million new cases in 2023 [1]. Recent advancements in medical science have led to the development of various cancer therapies, including surgery, radiotherapy, chemotherapy, and immunotherapy. Despite their efficacy in curbing tumor growth, these treatments often result in significant side effects, compromising therapeutic efficacy, prognosis, and increasing the likelihood of disease recurrence. In this context, nanoparticles (NPs) emerge as a promising alternative, offering stability, efficiency, and ease of modification as drug carriers. NP-based drug delivery systems (DDSs) enhance targeted drug delivery, minimize drug leakage, and facilitate sustained and controlled release. This contributes to prolonged bloodstream circulation and an improved therapeutic index.

However, drug-loaded NPs still encounter challenges related to targeting efficiency and biocompatibility [2]. Scientists have vigorously pursued active targeting strategies, aiming to precisely direct drugs by modifying NPs with target ligands (aptamers, peptides, antibodies, etc.). Regarding biocompatibility, NPs are often coated with various polymeric materials to serve as a “stealth” shield, preventing their detection by the immune system. One of the popular options is polyethylene glycol (PEG) polymers—an inert amphiphile known for its low cytotoxicity and high biocompatibility. By conjugating with PEG, NPs can dramatically lower immunogenicity and enhance the extracellular blood plasma half-life [3]. However, a study by Lubich et al. [4] indicated that repeated administration of PEG-conjugated NPs in rats led to accelerated blood clearance and a less advantageous biodistribution profile. As alternatives, other polymers, including dextran, chitosan (CS), polyvinyl pyrrolidone (PVP), and poly(lactic-co-glycolic acid) (PLGA), have also been employed as stealth coatings.

Among the various methods for NPs’ surface modification, biomimetic strategies are garnering increasing attention, due to their non-toxic and stealth properties, which draw inspiration from the concept of “learning from nature” [2,5]. Following the biomimetic strategy, cell membrane-coated nanoparticles (CMC@NPs) have been devised, forming a core–shell structure. This structure features NPs as the inner core (liposomes, inorganic particles, polymers, etc.) and a cell membrane coating as the external shell. NPs camouflaged with cell membranes inherit the ability to “disguise themselves”, enabling them to evade the reticuloendothelial system [6]. Furthermore, cell membranes sourced from various origins provide NPs with distinct biological functionalities. For example, neutrophil membrane-coated nanoparticles (Nm@NPs) are shown to home in on inflammation sites [7], while cancer cell membrane-coated nanoparticles (CCm@NPs) have demonstrated effective homotypic binding [8].

In this review, we aim to establish a comprehensive overview of CMC@NPs for tumor therapeutics. We begin by discussing the preparation methods, followed by an introduction to the features and applications of various cell membrane-based drug delivery systems. Following this, we highlight the notable benefits that CMC@NPs have shown in cancer treatment. Finally, we delve into the challenges and potential future trends in the application of CMC@NPs.

## 2. Classification of Cell Membrane-Coated Nanoparticles

According to the type of source cell membranes, CMC@NPs are mainly classified as red blood cell membrane-coated nanoparticles (RBCm@NPs), platelet membrane-coated nanoparticles (PLTm@NPs), white blood cell membrane-coated nanoparticles (WBCm@NPs), stem cell membrane-coated nanoparticles (SCm@NPs), CCm@NPs, and hybrid membrane-coated nanoparticles (Hym@NPs).

**Figure 1:**
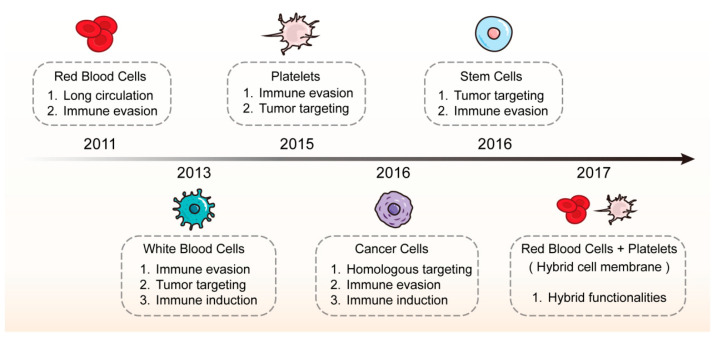
The representative development and features of CMC@NPs (created by the authors using Adobe Illustrator 2023^®^ software).

As shown in Figure 1, the first CMC@NPs were coated with red blood cell membranes (RBCm). In 2011, Hu et al. [9] prepared RBCm@NPs using a top-down method by extruding PLGA NPs with preformed RBCm-derived vesicles. The RBCm@NPs exhibited a circulation half-life of 39.6 h, significantly outperforming PEG-coated NPs, which had a half-life of 15.8 h. In 2013, Parodi et al. [10] developed WBCm@NPs, by coating nanoporous silicon microparticles with membranes purified from leukocytes. They discovered that white blood cell membrane (WBCm)-based NPs reduced phagocytic uptake, adhered to the inflamed endothelium, and enhanced drug accumulation at tumor sites. Building on the study of RBCm@NPs, Hu’s group introduced PLTm@NPs in 2015 [11], incorporating the platelet membrane with Doxorubicin (DOX)-loaded nanogels for breast cancer treatment. PLTm@NPs not only demonstrated prolonged circulation, but also showed exceptional targeting of cancer cells. In 2016, Rao et al. [12] developed CCm@NPs using upconversion NPs cloaked with cancer cell membrane (CCm)-derived vesicles for diagnosis and therapy. These NPs were uniquely capable of targeting tumor cells due to homologous adhesion and exhibited immune escape abilities. Concurrently, Gao et al. [13] introduced SCm@NPs, employing stem cell membranes (SCm) to wrap gelatin nanogels loaded with DOX, thereby enhancing their chemotherapeutic efficacy. The coated nanogels exhibited a high affinity for tumors and a reduced clearance via the reticuloendothelial system. In a pioneering study by Dehaini and co-workers [14] in 2017, the concept of Hym@NPs was introduced, combining RBCm and platelet membranes (PLTm) to cloak PLGA NPs. The Hym@NPs exhibited characteristics of both source cells.

Beyond the widely used option of cell membranes, several unconventional choices have emerged for coating NPs, such as bacterial membranes [15], viral-mimicking membranes [16], neural cell membranes [17], and fibroblast cell membranes [18], among others. These investigations open up additional avenues for the design of biomimetic NPs.

## 3. Preparation of Cell Membrane-Coated Nanoparticles

**Figure 2:**
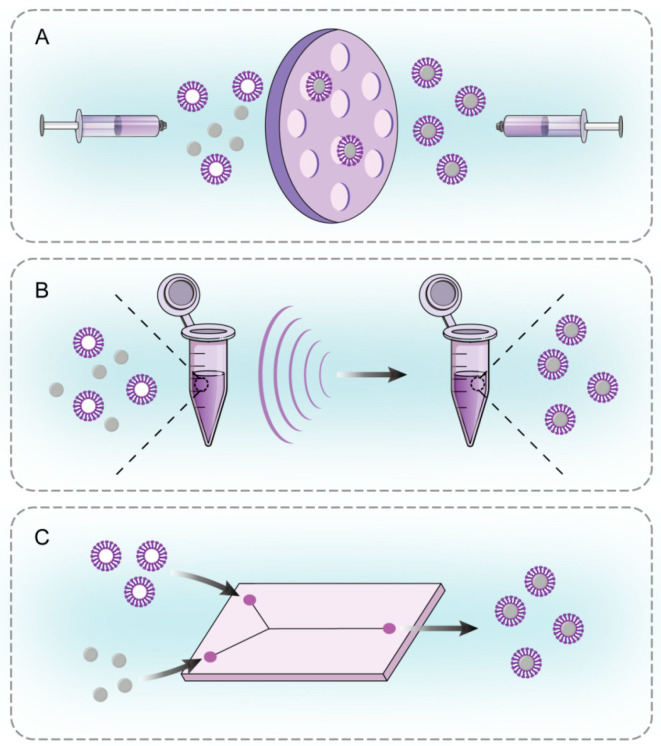
Preparation of CMC@NPs. (**A**) Co-extrusion. (**B**) Sonication. (**C**) Microfluidic electroporation (created by the authors using Adobe Illustrator 2023^®^ software).

Currently, the preparation of CMC@NPs is primarily conducted through a top-down approach. The initial step involves the isolation of cell membranes, where collected cells undergo hypotonic lysis [19], physical homogenization [20] or repeated freeze–thaw cycles [21] to disrupt the membrane. Subsequently, differential centrifugation is used to eliminate the intracellular contents, resulting in the collection of membrane fragments. These fragments can be resized into vesicles by extrusion through a polycarbonate membrane, if desired. The membrane fragments or vesicles are then coated onto the surface of NPs predominantly using co-extrusion, sonication, or microfluidic electroporation (Figure 2). Moreover, membrane coating can also occur spontaneously through electrostatic attraction between the charges of NPs and membrane vesicles [22].

### 3.1. Co-Extrusion

Co-extrusion is a common method for constructing CMC@NPs, where cell membrane vesicles and NPs are extruded through polycarbonate membranes with progressively smaller pore sizes. This process generates a force that can alter the membrane’s structure and allows it to reassemble on the surface of NPs. CMC@NPs created via co-extrusion not only demonstrate uniform distribution, but also effectively preserve the cell membrane proteins and their biological functions. Li et al. [23] produced RBCm@NPs by physically co-extruding Ag_2_S quantum dots (inner core) with erythrocyte vesicle membranes. While co-extrusion is popular in laboratory settings, scaling it up to large-scale production poses challenges due to its labor-intensive and time-consuming nature.

### 3.2. Sonication

Sonication stands out as both facile and time-efficient compared to co-extrusion, fusing membrane vesicles and nano-vehicles through ultrasonic waves. Chen et al. [24] innovatively cloaked mesoporous silica-coated bismuth nanorods with PLTm using sonication. In research by Cai et al. [25], Hym@NPs crafted via sonication exhibited enhanced stability and smaller dimensions, relative to those produced using co-extrusion. Achieving a uniform distribution and a thorough coating necessitates the optimization of several parameters, including amplitude, frequency, and duration. Furthermore, vigilant monitoring of potential temperature increases during sonication is essential, as they may cause the denaturation of the membrane proteins.

### 3.3. Microfluidic Electroporation

Microfluidic electroporation has gained increasing popularity among researchers since its conceptual demonstration in 2001. Microfluidic chips designed for electroporation offer enhanced transfection efficiency and reduced voltage requirements [26]. Each chip features two inlets, enabling the introduction and thorough mixing of NPs and membranes within the channel. As the mixture travels through the electroporation zone, electrical pulses create small pores in the membranes, facilitating the entry of NPs, with the resultant CMC@NPs collected at the outlet [27]. Wu et al. [28] successfully coated magnetic NPs with neutrophil vesicles using an electrophoresis microfluidic chip. This technique facilitates the high-throughput production of CMC@NPs, characterized by excellent encapsulation and high stability [29]. Despite its cost, microfluidic electroporation presents significant potential for practical applications.

## 4. Application of Cell Membrane-Coated Nanoparticles in Cancer Treatment

Over the past decade, CMC@NPs have been explored in various anti-tumor strategies, showcasing broad benefits and significant potential (Table 1). Here, we describe the properties and mechanisms of different classifications of CMC@NPs, including RBCm@NPs, PLTm@NPs, WBCm@NPs, SCm@NPs, CCm@NPs, and Hym@NPs and introduce recent advances in tumor therapy.

### 4.1. Red Blood Cell Membrane-Coated Nanoparticles (RBCm@NPs)

Red blood cells (RBCs), the most abundant cells in blood, primarily function to transport oxygen to organs and cells and can circulate in the bloodstream for up to an average of 120 days. Initially, erythrocytes were explored as carriers for transporting various bioactive compounds [96] and Hu et al. [9] were the first to extract the RBCm for biomimetic coating. With a concentration of approximately 5 billion per milliliter in the blood, erythrocytes offer plentiful material for isolation, facilitated by their lack of a nucleus and complex organelles.

RBCm stand out as a promising biomimetic material for enhancing the residence time of NPs in the bloodstream. This advantage is attributed to the “self-marker” proteins on the membrane surface, enabling the NPs to evade the reticuloendothelial system. CD47 is a key marker that interacts with the inhibitory receptor SIRPα (a signal-regulating protein present on macrophages) and is recognized as a “do not eat me” signal, thus shielding RBCm@NPs from phagocytosis [6,97]. Owing to their capabilities for immune evasion and prolonged circulation, RBCm@NPs have been extensively researched for anti-tumor drug delivery, as detailed in Table 2. However, their clinical application is constrained by poor targeting [6]. Fang et al. suggested a lipid insertion method to enhance the tumor-targeting efficiency of cell membrane-based NPs without affecting protein biodistribution, where target ligands such as folic acid, tumor-targeting peptides, or neurotoxin-derived peptides are integrated into CMC@NPs as ligand–linker–lipid conjugates through lipid tethers.

**Figure 3:**
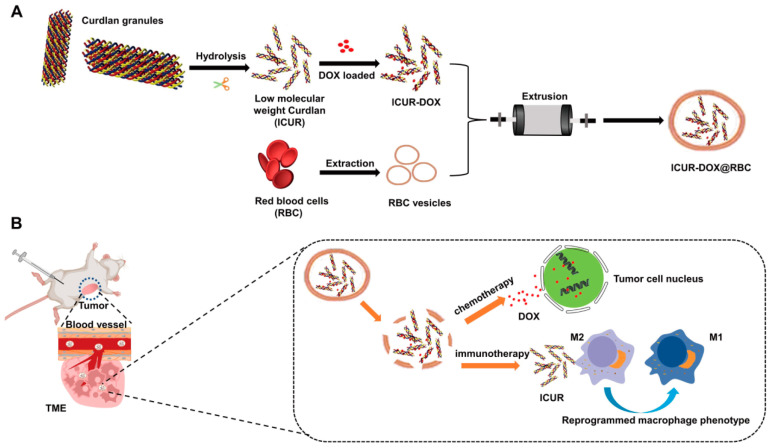
(**A**) The synthetic route for the lCUR-DOX@RBC. (**B**) Schematic illustration of chemo-immunotherapy of lCUR-DOX@RBC in tumor-bearing mice. Reprinted with permission from Ref. [31]. Copyright © 2024, Elsevier B.V: Amsterdam, The Netherlands.

Immunotherapy suppresses tumor proliferation by restoring normal immune responses and, when synergized with chemotherapy, it demonstrates increased anticancer efficacy. Curdlan, a non-branched β-glucan, activates receptors on macrophages to induce the production of pro-inflammatory cytokines [98] and transforms tumor-associated macrophages (TAMs) into an inflammatory phenotype [99]. Low molecular weight curdlan (lCUR) served as an immunomodulatory drug carrier in research conducted by Lin and co-workers [31]. They engineered RBCm-coated lCUR loaded with DOX for melanoma treatment (lCUR-DOX@RBC), as illustrated in Figure 3. To evaluate the immune evasion capability of RBCm coatings, DOX, lCUR-DOX, and lCUR-DOX@RBC were separately incubated with bone marrow-derived macrophages (BMDMs), with the least fluorescence observed in lCUR-DOX@RBC-treated samples, indicating reduced macrophage uptake. In vivo studies showed that lCUR-DOX@RBC achieved prolonged blood circulation and significantly enhanced tumor growth inhibition after 12 days of treatment.

Photodynamic therapy (PDT) and photothermal therapy (PTT), viewed as promising tumor therapeutic approaches, boast broad applicability, non-invasiveness, and straightforward implementation [100]. Melanin, known for its excellent PTT properties, has been identified as a potent agent [101]. Zhang et al. [36] extracted melanin NPs (HNPs) from human black hair for liver cancer PTT. Modified with the cRGD peptide via the lipid insertion approach, RBCm was coated onto HNPs (HNP@RBCm-cRGD) to enhance their circulation time and achieve a greater accumulation efficiency, without compromising the PTT conversion capability of HNP.

### 4.2. Platelet Membrane-Coated Nanoparticles (PLTm@NPs)

Platelets (PLTs) are derived from the cytoplasmic fragmentation of mature megakaryocytes and represent the smallest cells in the blood. PLTs have a complete cell membrane structure but lack a nucleus, facilitating the isolation of the PLTm. Similar to RBCm@NPs, the inclusion of the CD47 protein on the modified PLTm coating helps to prevent phagocyte detection. Moreover, PLTm are endowed with a range of functional antigens, receptors, and proteins, such as CD55, CD59, P-selectin, and glycoprotein (GP) Ib, which are instrumental in tumor targeting and metastasis, bacterial infections, thrombosis, and more [11].

**Figure 4:**
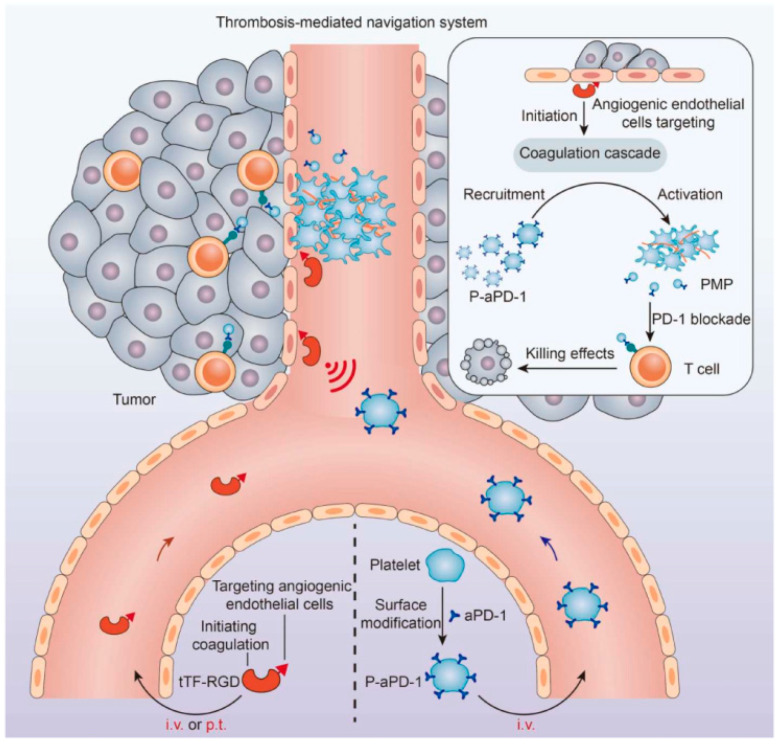
Schematic of the thrombosis-mediated navigation system for P-aPD-1. Reprinted with permission from Ref. [43]. Copyright © 2024 The Authors, some rights reserved.

The applications of PLTm@NPs in cancer therapy are detailed in Table 3. Bahmani et al. [42] crafted polylactic acid (PLA) cores encapsulating resiquimod (R848), a Toll-like receptor (TLR) agonist, and then coated these cores with PLTm, exploiting the ability of PLTm to selectively bind to cancer cells in the tumor microenvironment (TME). PLTm@NPs have the capability to respond to thrombotic signals that attract PLTs. This observation led Wang and coworkers [43] to devise a strategy that utilizes specific coagulation to navigate drug delivery to the tumor site and boost anti-tumor efficacy. They formulated a fusion protein, truncated tissue factor-Arg-Gly-Asp (RGD) (tTF-RGD), as a catalyst to trigger the coagulation cascade, and anti-PD-1 antibody-conjugated PLTs (P-aPD-1) that react to coagulation signals. When tTF-RGD was administered via either the intravenous (i.v.) or peritumoral (p.t.) route, it enhanced coagulation signals at the tumor site, attracting P-aPD-1. PLTs activated by the coagulation cascade then secrete platelet-derived microparticles (PMPs) and discharge aPD-1 antibodies to rejuvenate T cells (Figure 4). In this thrombosis-mediated navigation, maintaining moderate coagulation is critical, as excessive coagulation could pose risks.

PLTm@NPs are utilized to deliver photosensitizers or photothermal agents. Xu et al. [40] created a PLTm coating on photosensitizer (verteporfin)-preloaded PLGA NPs (NP-Ver@P). NP-Ver@P exhibited a significantly higher tumor uptake compared to their RBCm-coated counterparts, yet maintained a similar systemic circulation. In subsequent research, PLT vesicles were engineered with Cu_2_O NPs encapsulating an aggregation-induced emission (AIE) photosensitizer (TBP-2), facilitating a prolonged blood circulation and an improved tumor targeting capability, as demonstrated by Ning and co-workers [41]. They introduced a cuproptosis sensitization system that triggers multiple tumor cuproptosis inductions, effectively suppressing lung metastasis of breast cancer in both cell lines and mouse models.

In addition to cancer therapy, PLTm@NPs are broadly applied in disease diagnosis and treatment, leveraging the unique biological functions of PLTm. These include applications in conditions like atherosclerosis, thrombotic, acute kidney injury, hypoxic pulmonary hypertension, and intracerebral hemorrhage, among others.

### 4.3. White Blood Cell Membrane-Coated Nanoparticles (WBCm@NPs)

White blood cells (WBCs), also known as leukocytes, play a pivotal role in recognizing and eliminating pathogens, while regulating the immune system, which is essential for maintaining physiological function. These cells are drawn to inflammation sites by chemokines and since chronic inflammation is a hallmark of cancer—with an abundance of chemokines being overexpressed—it leads to the targeted accumulation of WBCm@NPs in tumor tissues [105]. Furthermore, specific immune recognition proteins on the WBCm can bind to molecules on cancer cells, offering precise site-specific targeting [106]. Additionally, NPs camouflaged with WBCm have the ability to evade immune detection. These capabilities position WBCm@NPs as a promising anticancer drug delivery system, exemplified by Nm@NPs, macrophage membrane-coated NPs (Mm@NPs), T cell membrane-coated NPs (Tm@NPs), dendritic cell membrane-coated NPs (DCm@NPs), and natural killer cell membrane-coated NPs (NKm@NPs). The applications of WBCm@NPs in cancer therapy are cataloged in Table 4.

#### 4.3.1. Neutrophil Membrane-Coated Nanoparticles (Nm@NPs)

Differentiating from hematopoietic stem cells (HSCs) in bone marrow, neutrophils represent the most prevalent type of WBCs in peripheral blood, comprising 55–70% of the total and serving a crucial role in pro-inflammatory reactions and immune responses to pathogens. Chemokines, such as the CXC chemokine family, summon neutrophils to inflamed joints to mitigate inflammation and facilitate tissue repair [110]. Capitalizing on this mechanism, Yang et al. and Zhang et al. [111,112] developed NPs coated with neutrophil membranes (Nm) for precise drug delivery, aimed at rheumatoid arthritis therapy. Additionally, Zhang et al. [113] engineered antibiotic-loaded Nm@NPs coupled with natural microalgae for antibiotic delivery in the lungs, significantly reducing mortality with minimal toxicity in a mice model.

In the management of pancreatic diseases, the presence of the blood–pancreas barrier often results in a limited drug distribution at pancreatic sites. Coating NPs with Nm enables them to overcome this barrier, as shown by Cao et al. [47]. Nm-coated PEG-PLGA NPs were utilized to deliver celastrol for maximum internalization in the pancreas, exhibiting significant anti-tumor activity in both ectopic and orthotopic models.

**Figure 5:**
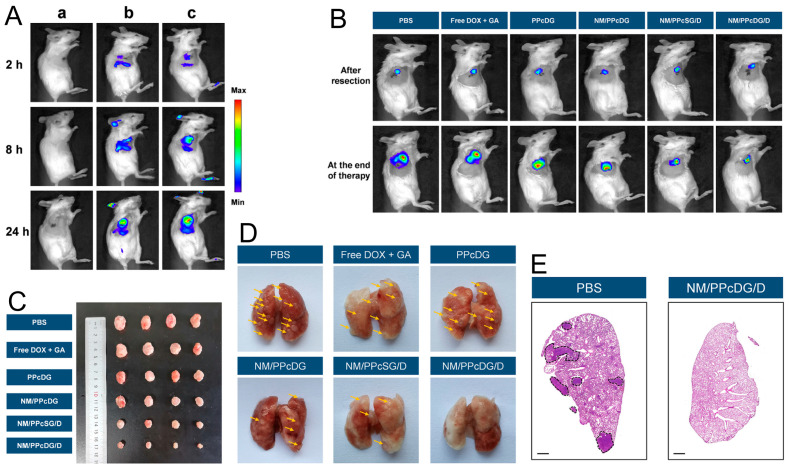
(**A**) In vivo living images after various treatments in tumor-excising mice. (**a**), PBS; (**b**), PPcDG/DiD; (**c**), NM/PPcDG/DiD. (**B**) In vivo imaging of tumors after tumor resection and at the end of therapy. (**C**) Typical tumor images in various groups at the end of therapy. (**D**) Representative pictures of lung tissues in various groups at the end of therapy (some metastatic nodules were in the back). (**E**) Full scanning of the H&E staining sections of the lung tissues in the PBS group and the NM/PPcDG/D group. Black circles indicated the typical metastasis in the PBS group (Scale bar: 1000 μm). Reprinted with permission from Ref. [7]. Copyright © 2024 Acta Materialia Inc.: Oxford, UK.

Nm@NPs have been explored in cancer treatment experiments for their potential to suppress tumor growth and metastasis. Xia and co-workers [7] developed a sponge-like Nm-coated nanocarrier (NM/PPcDG/D), aimed at disrupting postoperative inflammatory immunosuppressive areas and blocking the formation of pulmonary pre-metastasis niches by reducing the infiltration of myeloid-derived suppressor cells to boost treatment efficacy for metastasis and relapse. In vivo imaging revealed that the NM/PPcDG/D group achieved a higher accumulation rate at the post-operative tumor region than PPcDG/D, demonstrating a significant inflammatory tropism. Furthermore, it was noted that the NM/PPcDG/D-treated group significantly inhibited tumor recurrence and presented fewer metastatic nodules in lung tissues (Figure 5).

Circulating tumor cells (CTCs) are the generic term for tumor cells shed from primary solid tumors that enter the blood circulation [114]. Detecting CTCs is important for early tumor diagnosis, as well as for predicting recurrence and metastasis, and the required blood samples are more convenient and non-invasive than other biopsy strategies. Wu et al. [28] developed Nm-coated immunomagnetic NPs to ensure the targeting ability for CTCs in blood, resulting in significant improvements in both the efficiency and purity of CTC isolation.

#### 4.3.2. Macrophage Membrane-Coated Nanoparticles (Mm@NPs)

Macrophages, which derive from HSCs and mature from monocytes, have the capability to survive for months, or even longer. They play a key role in the innate immune response and protect the body by phagocytosing microorganisms, apoptotic cells, and presenting antigens [115]. The adhesion molecules expressed on macrophages’ surface, such as integrins, selectins, and antigens, facilitate adhesion to inflammatory and cancer cells [116]. Consequently, macrophage membrane (Mm)-coated NPs can specifically target tumor and inflammatory tissues, bypass immune system clearance, achieve prolonged circulation in the blood, and, additionally, activate anticancer immunity.

The application of Mm@NPs for anti-tumor therapies is chiefly concentrated on the TME and TAMs. The TME represents a complex, integrated system, resulting from the interaction of tumor cells with surrounding tissues and immune cells, contributing to tumor malignant progression, immunosuppression, drug resistance, and invasive metastasis [117]. It is characterized by conditions such as hypoxia, reductive states, acidic pH, and hydrogen peroxide (H_2_O_2_) overexpression, which foster tumorigenesis and proliferation [118]. Building on this understanding, Wen and co-workers [119] developed an Mm-coated mesoporous silica nanoplatform, loaded with catalase, DOX, and R848. This platform is capable of producing oxygen on-site, inducing immunogenic cell death (ICD), and boosting dendritic cells’ activity, respectively, to amplify the immunotherapeutic efficacy. Experimental results showed that the immunosuppressive TME could be mitigated by oxygen generation in the tumor area, suggesting that integrating it with immunotherapy offers a viable strategy for combating tumors.

**Figure 6:**
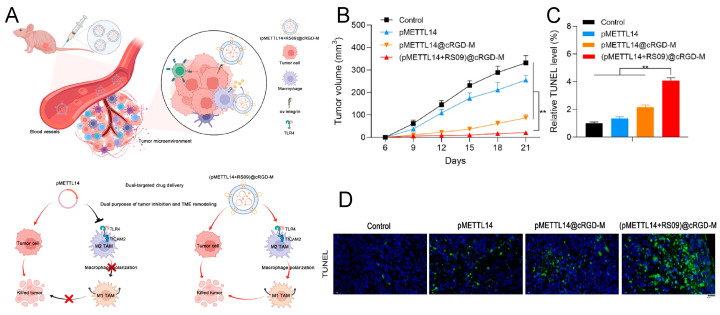
(**A**) Schematic illustration of (pMETTL14+RS09)@cRGD-M in dual-targeted tumor therapy. (**B**) Tumor volumes of different groups (** *p* < 0.01). (**C**,**D**) TUNEL staining indicated cell apoptosis in tumor tissues (Scale bar: 20 μm, ** *p* < 0.01). Reprinted with permission from Ref. [51]. Copyright © 2024 The Authors.

As a major component of the TME, TAMs can be polarized into two phenotypes, as follows: anti-tumor M1 type and pro-tumor M2 type. Polarizing TAMs from M2 to M1 is an effective strategy to remodel the TME for tumor treatment. TLR agonists have been reported to induce polarization of TAMs to M1 [120]. Huang et al. [51] created (pMETTL14+RS09)@cRGD-M, an Mm-coated nanovesicle modified with the cRGD peptide, to co-deliver the TLR4 agonist and anti-cancer drug (METTL14), aimed at tumor inhibition and TME remodeling. The nanovesicles, assisted by Mm, can dual-target tumors and macrophages, and cRGD modification enhances their targeting ability. After 21 days of treatment in mice, (pMETTL14+RS09)@cRGD-M showed excellent anti-tumor effects (Figure 6). In another study, Yue et al. [108] developed Mm-coated NPs with a polydopamine core, which serves as a photothermal transduction agent, to carry TMP195 (an epigenetic modulator that repolarizes M2 into M1), targeting the cancer area after PTT through inherited inflammation-mediated chemotaxis from Mm, enhancing the tumor elimination rate.

**Figure 7:**
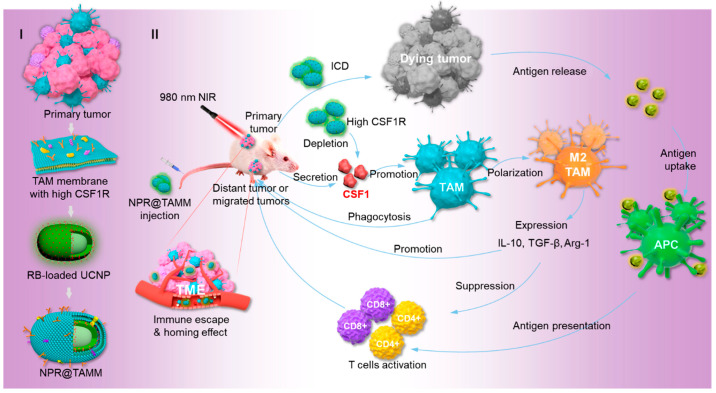
Schematic illustration of the TAM-membrane-coated upconversion NPs for improved photodynamic immunotherapy. (I) Preparation of NPR@TAMM. (II) Schematic representation of antitumor mechanism by NPR@TAMM. Reprinted with permission from Ref. [50]. Copyright © 2024 American Chemical Society: Washington, DC, USA.

Tumor cells secrete macrophage colony stimulating factor 1 (CSF1), which binds to the receptor (CSF1R) on TAMs, leading to polarization towards the M2 immunosuppressive phenotype [121]. Chen et al. [50] isolated a TAM membrane (TAMm) and enveloped it around upconversion NPs with a conjugated photosensitizer (NPR@TAMM) to integrate PDT and immunotherapy in cancer treatment. As shown in Figure 7, the TAMm coating mimics the source cells, binding with CSF1 to counteract the polarization of M2, and enhances drug delivery to tumor sites through its homing ability to TAMs, thereby boosting anti-tumor immune efficacy. Similarly, Song et al. [49] developed an M1 membrane-coated magnetic PTT nanocore for immunotargeting and photoacoustic imaging-guided cancer treatment.

#### 4.3.3. T Cell Membrane-Coated Nanoparticles (Tm@NPs)

T cells, a type of lymphocyte originating from lymphoid stem cells, participate in the innate immune system [122]. They have multiple receptors that specifically identify and bind to tumor-derived antigens on the membrane [123], which allows them to eliminate tumor cells directly or by producing various growth factors and cytokines, thereby enhancing immune efficacy. Due to the high tumor affinity of T cell receptors, Tm@NPs represent a promising nano-platform for targeted tumor drug delivery.

Tm@NPs are capable of devastating cancer cells in a way that resembles cytotoxic T lymphocytes (CTLs). Kang et al. [54] developed T cell-mimicking NPs by creating PLGA NPs and loading them with anticancer drugs, such as dacarbazine. Tm@NPs actively target tumor cells through adhesion proteins, eliminating cells by generating a Fas ligand signal and releasing drugs. They also restore CTL functions by blocking immune checkpoint interactions and scavenging for immunosuppressive molecules. In a B16F10 tumor-bearing mouse model, it was observed that Tm@NPs were more effective in retarding tumor growth with negligible systemic toxicity, especially when compared to immune checkpoint blockade (anti-PD-L1 antibody) treatment.

**Figure 8:**
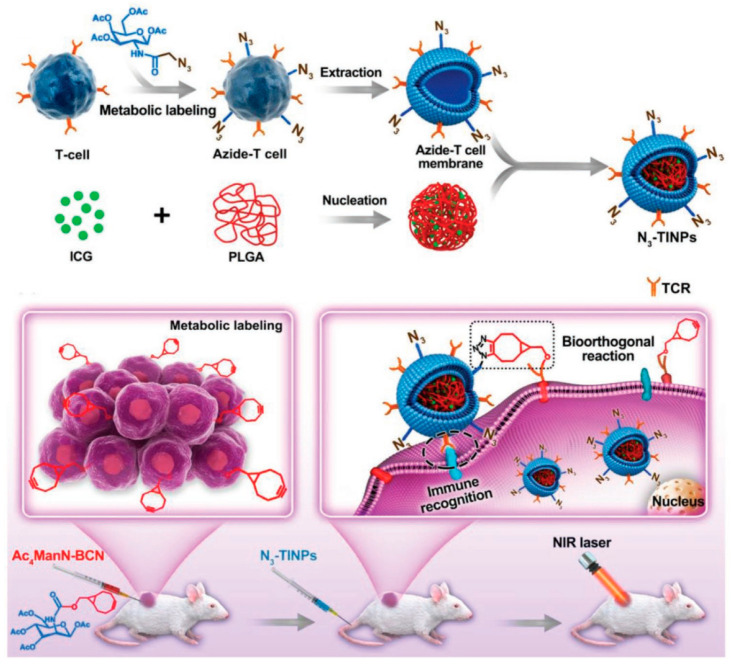
Fabrication of N_3_-TINPs and their application for highly efficient photothermal therapy. Reprinted with permission from Ref. [57]. Copyright © 2024 The Authors.

To combat the inter- and intra-heterogeneity of tumors, researchers have adopted a dual-targeting strategy for more effective drug delivery to malignant sites. As reported, Ac_4_ManN-BCN (a novel [6.1.0] bicyclo nonyne-modified unnatural sugar) can be artificially introduced into various tumor cells [124]. Han et al. [57] modified T cell membrane (Tm) with the azide (N_3_) through glycometabolism, enveloped it in PLGA polymeric cores loaded with indocyanine green (ICG), and developed N_3_-TINPs for PTT. As depicted in Figure 8, N_3_ anchors to tumor cells previously administered with BCN-sugar, Ac_4_ManNBCN, via a bioorthogonal reaction, while Tm targets the tumor through retained immune recognition receptors, achieving dual targeting. Subsequently, tumors are eradicated through ICG-mediated PTT.

In a subsequent experiment, chimeric antigen receptor T (CAR-T) cells were engineered to enhance targeting efficiency. Ma et al. [55] created mesoporous silica NPs containing IR780, encased in a CAR-T cell membrane. This membrane is specifically designed to recognize Glypican-3 expression in hepatocellular carcinomas (HCCs), thereby enabling targeted PTT treatment.

**Figure 9:**
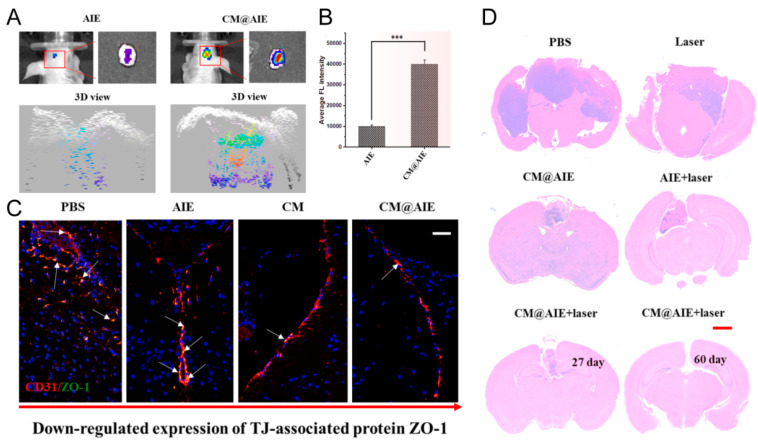
(**A**,**B**) In vivo fluorescence imaging quantitative fluorescence intensity analysis after Cy5.5-labeled AIE NPs and CM@AIE NPs administrations, respectively (*** *p* < 0.001). (**C**) Immunofluorescence images of ZO-1 and CD31 on brain blood vessels after various treatments for 8 h (Scale bar: 100 μm). (**D**) H&E analysis of tumor slices after various treatments (Scale bar: 50 μm). Reprinted with permission from Ref. [56]. Copyright © 2024 Elsevier Ltd.: Oxford, UK.

Wang et al. [56] specifically developed Tm@NPs with AIE (CM@AIE) for brain cancer theranostics. Through genetic engineering, Tm was tailored to bind to CD133-positive glioblastoma stem cells and epidermal growth factor receptors, targeting both GSCs and glioblastoma cells. In vivo fluorescence imaging showed that mice injected with Cy5.5-labeled CM@AIE exhibited a significantly brighter fluorescence in the GBM area compared to bare AIE NPs, with the tumor signal being nearly four times stronger, highlighting Tm’s exceptional targeting capability (Figure 9). Zonula occludens-1 (ZO-1), crucial for maintaining tight junction (TJ) structures and integrity, was found to be down-regulated in tumor blood vessels following Tm and CM@AIE treatment, suggesting Tm’s ability to disrupt TJ tightness and silently cross the blood–brain barrier (BBB). CM@AIE effectively inhibited GBM proliferation through 980 nm laser irradiation (0.70 W/cm², 10 min), resulting in no noticeable tumor cell growth in the group that had been intravenously injected with CM@AIE after 60 days.

#### 4.3.4. Dendritic Cell Membrane-Coated Nanoparticles (DCm@NPs)

Dendritic cells (DCs) are pivotal antigen-presenting cells (APCs), essential for inducing and regulating innate and adaptive immune responses in the human body [125]. DCs can recognize, engulf, and modify foreign antigens; present these antigens through major histocompatibility complexes (MHCs); and, subsequently, activate various T lymphocyte subtypes to target tumor cells. However, the TME can promote the emergence of tolerogenic DCs that aid tumor immune evasion by either inducing anergy and apoptosis in CD8^+^ T cells or activating regulatory T cells [126]. Consequently, the development of DC-mimicking NPs, also known as nano-vaccines, capable of antigen processing, T cell priming, and stimulating anti-tumor immunity, is garnering growing interest.

**Figure 10:**
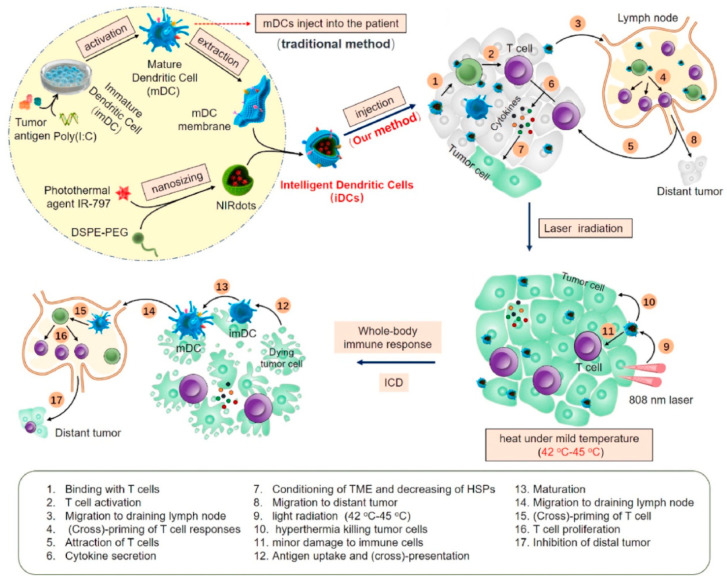
Schematic illustration of the preparation of iDCs and the mechanism of synergy between iDCs and mild photothermal-immunotherapy. Reprinted with permission from Ref. [61]. Copyright © 2024 Elsevier Ltd.: Oxford, UK.

Sun et al. [61] cultivated immature DCs with tumor antigens for 24 h, utilizing TLR-3 to induce their maturation, followed by harvesting the mature dendritic cell membrane (DCm). Intelligent DCs (iDCs) were then synthesized by coating NPs, which carried PTT agents, with DCm, to create a synergistic PTT–immunotherapy approach for anti-tumor effects. iDCs are capable of stimulating T cells both in situ and in lymph nodes. Additionally, cytokines secreted by activated CD4^+^/CD8^+^ T cells enhance the sensitivity of cancer cells to heat stress. With 808 nm laser radiation for mild PTT therapy (42–45℃), tumor cells, sensitized to the treatment, were efficiently eradicated while sparing immune cells, thereby reactivating the tumor immunity cycle (Figure 10).

**Figure 11:**
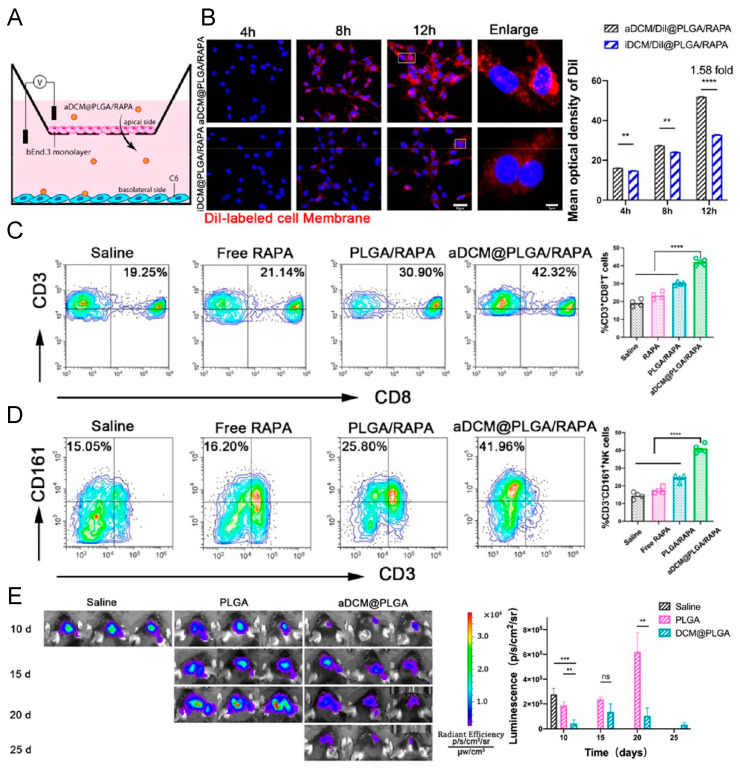
(**A**) Transwell schematic illustrations. (**B**) Confocal images. (**C**,**D**) Flow cytometric quantification of T and NK cells expressed by brain tumor tissue (**** *p* < 0.0001). (**E**) In vivo imaging system detection images and bioluminescence quantification of mice preimmunized with different formulas (** *p* < 0.01, *** *p* < 0.001). Reprinted with permission from Ref. [63]. Copyright © 2024 American Chemical Society: Washington, DC, USA.

Xu et al. [60] developed DCm-coated photosensitizer (AIE) lipid droplets to traverse biological barriers and efficiently accumulate at tumor sites. Additionally, DCm@NPs demonstrated significant potential in inhibiting glioma. Ma et al. [63] engineered RAPA-loaded PLGA encapsulating DCm (with DCs induced to mature in vitro), termed aDCM@PLGA/RAPA. As depicted in Figure 11, using a transwell BBB model, it was observed that aDCM@PLGA/RAPA effectively crossed the BBB, evidenced by a higher fluorescence intensity in C6 cells after crossing the bEnd.3 monolayer. Following 19 days of treatment, enhanced CD3^+^CD8^+^ and CD3^+^CD161^+^ expression were quantified in brain tumors, indicating an increased T cell and NK cell activity, as shown using flow cytometry in the aDCM@PLGA/RAPA group. Moreover, aDCM@PLGA elicited a robust memory effect; post pre-immunization with various groups and subsequent injection of C6-LUC cells, minimal tumor volume was detected in the aDCM@PLGA group through bioluminescence imaging, in contrast to saline and PLGA.

#### 4.3.5. Natural Killer Cell Membrane-Coated Nanoparticles (NKm@NPs)

Natural killer cells (NKs), functioning as lymphocytes of the innate immune system, have the ability to recognize and eliminate aberrant cells, without prior antigen stimulation, a capability distinct from that of T cells. Contrarily, NKs can promote the maturation of APCs and secrete a variety of cytokines to modulate immune responses [127]. It is reported that CMC@NPs formulated with natural killer cell membrane (NKm) enable NPs to specifically target tumors and to induce and enhance M1 macrophage polarization, thereby producing anti-tumor immunity [64,109].

NKm@NPs are utilized to transport photosensitizers or anticancer drugs for brain cancer due to their ability to cross the BBB. Deng et al. [67] developed NKm coated with a polymeric core carrying AIE, while Zhang et al. [66] encapsulated cRGD-decorated NKm with PLGA-coated temozolomide and interleukin-15 (IL-15) NPs. Both delivery systems provided a highly efficient BBB crossing and performed well in inhibiting brain tumor growth.

**Figure 12:**
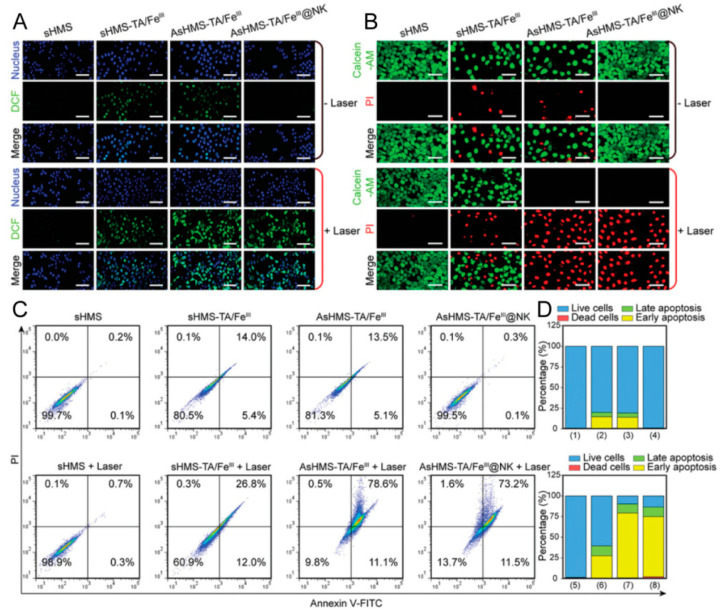
(**A**) Intracellular free radical generation via DCFH-DA assay. (**B**) Live/dead cell staining via calcein-AM/PI assay. (**C**,**D**) Apoptosis assay via Annexin V-FITC/PI staining. Reprinted with permission from Ref. [65]. Copyright © 2024 The Authors.

Free radical-based anti-tumor therapy, which induces tumor cell death through oxidative stress, is widely utilized [128]. However, glutathione, the primary endogenous antioxidant, can neutralize free radicals in tumor cells. Employing a photothermal-augmented thermodynamic–chemodynamic approach, Lin et al. [65] developed a nanogenerator (AsHMSTA/Fe^III^@NK) that both generates free radicals and consumes intracellular glutathione (GSH). This approach effectively amplifies oxidative stress, thereby enhancing anticancer therapy. As illustrated in Figure 12, an increased green fluorescence emission was noted in the AsHMS-TA/Fe^III^@NK group with NIR laser irradiation, relative to other groups, indicative of elevated free radical production. Further, live/dead cell staining and apoptosis analyses confirmed the AsHMS-TA/Fe^III^@NK nanogenerator’s significant efficacy in promoting tumor cell death predominantly via apoptosis.

### 4.4. Stem Cell Membrane-Coated Nanoparticles (SCm@NPs)

Stem cells (SCs), also known as mesenchymal stem cells (MSCs), offer significant advantages due to their ease of acquisition and isolation and their ability to be derived from a variety of tissue types [129]. MSCs are inherently capable of evading the immune system, due to their low immunogenicity [130], and they have the ability to migrate to malignant tissues, including glioma, breast cancer, and HCCs, through chemokine–receptor interactions and endothelial adhesion [131]. Consequently, MSC membrane-coated nanoparticles (MSCm@NPs) demonstrate substantial potential in anti-tumor applications. A list of SCm@NPs utilized in cancer therapy is detailed in Table 5.

Yang et al. [68] developed DOX-loaded PLGA NPs coated with MSC membranes (MSCm) derived from umbilical cord MSCs, aimed at targeted chemotherapy delivery for tumors. This study was the first to demonstrate the feasibility of using umbilical cord MSCm in creating nanocarriers. Following this, Zhang’s group [69] employed umbilical cord-derived MSCm@NPs for the chemo–photothermal treatment of malignant bone diseases. These MSCm@NPs retained the biological function of natural MSCm, exhibiting remarkable biocompatibility, stability, and synergistic efficacy in cancer cell eradication. Furthering this research, Mu’s group [70] engineered MSCm@NPs to deliver DOX and PD-L1 siRNA for chemoimmunotherapy of prostate tumor bone metastases. Their work demonstrated outstanding efficacy in both in vitro and in vivo studies.

Cell–cell interactions play a crucial role in both tissue regeneration and tumorigenesis, from development to maturation [132]. Drawing inspiration from this, Kim et al. [133] developed SCm-derived NPs (referred to as CMNPs), which incorporate a Notch-1 antagonistic aptamer for tumor angiogenesis suppression. These CMNPs aim to provide the following dual benefits: counteracting laryngeal cancer and promoting chondrogenesis. CMNPs were sourced from tonsil-derived MSCs, isolated using a series of filters with progressively smaller pore sizes. These NPs enhance cell–cell interactions and facilitate a tighter membrane-to-membrane contact, leading to a hypoxic environment conducive to both cartilage growth and cancer cell proliferation. Given that blood vessels are vital for cancer growth but unnecessary for cartilage development, the presence of the Notch-1 aptamer in CMNPs ultimately results in the death of laryngeal cancer cells and the promotion of chondrogenesis.

In another study, Sancho-Albero et al. [134] utilized the natural biogenesis pathway to obtain small extracellular vesicles (sEVs) loaded with NPs derived from SCs. These sEVs were employed in a multinodular intraperitoneal model to evaluate their selective delivery capabilities. The findings revealed that sEVs exhibited a high delivery efficacy, even in scenarios of multinodular dissemination, heralding a new avenue for the advancement of biomimetic NPs.

**Table 5:** List of SCm@NPs applied in cancer therapy.

Source Cell	Inner Cores	Therapeutics	Key Functions	References
Stem cells	PLGA	Chemotherapy	Tumor targeting	[68]
	Polydopamine	Chemotherapy–PTT	Tumor targeting, long circulation, immune evasion	[69]
	Polydopamine	Chemotherapy, gene therapy	Tumor targeting	[70]
	Fe_3_O_4_@PDA	MRI, PTT, gene therapy	Tumor targeting	[135]

### 4.5. Cancer Cell Membrane-Coated Nanoparticles (CCm@NPs)

Utilizing CCm to fabricate CCm@NPs for homologous cancer therapy presents substantial advantages. These NPs leverage tumor homotypic binding for specific targeting and efficient internalization by the originating tumor cells. This ability stems from the overexpression of homotypic adhesion antigens on CCm surfaces, including the E-cadherin, Galectin-3, and TF antigens [136]. CCm’ low immunogenicity permits the NPs to avoid immune detection, thus prolonging their circulation in the bloodstream. Furthermore, modifying these NPs with cancer antigens enhances immune responses, which is beneficial for immunotherapy. Additionally, the ease of proliferating tumor cells in vitro cell culture supplies ample resources for CCm@NP development [137]. As a targeted drug delivery platform, CCm@NPs have been employed across various therapeutic strategies against cancer. A list of CCm@NPs used in cancer therapy is provided in Table 6.

**Figure 13:**
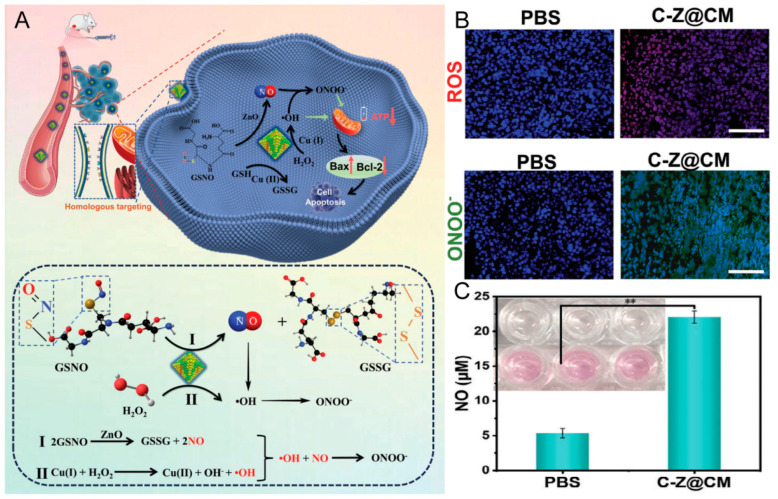
(**A**) Schematic illustration of C-Z@CM multi-mode anti-tumor actions and the main process and chemical equation of ONOO^−^ production. (**B**) Representative ROS and ONOO^−^ staining images of tumor tissues after different treatments for 24 h (Scale bar: 100 μm). (**C**) Quantitative analyses of NO content in tumor tissue (** *p* < 0.01). Reprinted with permission from Ref. [74]. Copyright © 2024 Wiley-VCH GmbH.

Wang et al. [81] created mesoporous silica nanorods with colorectal CCm@NPs (C-Z@CM), demonstrating a superior cellular uptake compared to uncoated counterparts in HT-29 cells. A powerful anti-tumor effect has been demonstrated with nitric oxide (NO) gas therapy. In Xu and colleagues’ research [74], as depicted in Figure 13, a copper-based metal–organic framework (MOF) material was synthesized. This material triggers the generation of hydroxyl radicals (•OH), with ZnO doping enhancing NO production from endogenous S-nitrosoglutathione (GSNO) at tumor sites. The concurrent release of reactive oxygen species (ROS) and NO led to the formation of lethal reactive nitrogen species (ONOO^-^). Treated with C-Z@CM, treatment in a U14 tumor-bearing mouse model resulted in markedly elevated ROS, NO, and ONOO^-^ levels.

Immunotherapy, combined with other treatments such as magnetic resonance imaging (MRI), sonodynamic therapy (SDT), PTT, and chemotherapy in CCm@NPs, has gained traction in anti-tumor therapies. Yang et al. [71] synthesized a nano-vaccine by coating B16-F10 melanoma membrane onto mesoporous silica NPs and incorporating adjuvant cytosine–phosphate–guanine (CpG) and propranolol (a β-AR inhibitor). This approach enhanced T cell infiltration into tumors and modulated the immunosuppressive TME. Shang et al. [8] developed a chemo-immunotherapy nanosystem by coating a pH-responsive nanogel loaded with paclitaxel (PTX) and interleukin-2 (IL-2) onto CCm, targeting triple-negative breast cancer. This strategy facilitated an increased drug concentration and a swift release at the target site, leading to rapid immune activation and amplified anti-tumor efficacy. In a separate study, Liu et al. [72] created DBE@CCNPs, incorporating polyethylenimine (PEI25K) containing unmethylated CpG into a CCm modified with CD47KO/CRT. They utilized gene editing to produce dual-bioengineered tumor cells, cultivated with calreticulin to induce ICD, enhancing antigen phagocytosis. The integration of this nano-vaccine with anti-PD-L1 therapy in a mouse model effectively counteracted T lymphocyte immunosuppression, resulting in a significant anti-tumor response.

Fu et al. [79] developed a nanoplatform for MRI-guided immuno-chemodynamic therapy of osteosarcoma, comprising hollow manganese dioxide (HMnO_2_) NPs modified with alendronate and coated with CCm to encapsulate ginsenosides Rh2 (immune activators). Upon internalization by tumor cells via homophilic targeting, the HMnO_2_ framework depletes GSH and generates •OH, effectively inducing ICD and activating T cells to mitigate the tumor. In their study, Jiang et al. [76], introduced a CCm-coated, catalase-mimicking, Fe-doped polydiaminopyridine (Fe-PDAP) containing chlorin e6 (Ce6), termed MFC, for efficient SDT-immunotherapy. MFC minimizes unnecessary H_2_O_2_ consumption and proficiently generates ROS at the tumor site, enhancing the release of tumor-associated antigens during SDT, to promote the maturation of APCs and trigger an immune response.

**Figure 14:**
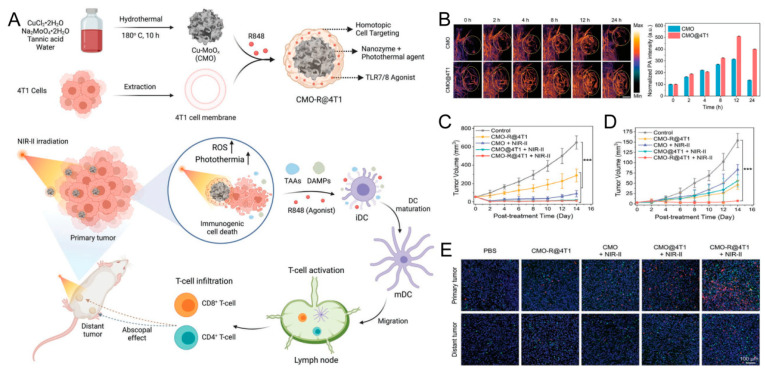
(**A**) Scheme of CMO-R@4T1-mediated targeted NIR-II photothermal immunotherapy. (**B**) In vivo photoacoustic imaging and intensity of tumor-bearing mice with the tumor regions highlighted (yellow circle) (Scale bar: 5 mm). (**C**,**D**) Tumor growth curves of primary and distant tumors on bilateral tumor-bearing mice after various treatments (*** *p* < 0.001). (**E**) Immunofluorescence staining of CD8^+^ T cells (green) and CD4^+^ T cells (red) from both primary and distant tumor tissues of mice after different treatments (Scale bar: 100 μm). Reprinted with permission from Ref. [77]. Copyright © 2024, Wiley-VCH: Weinheim, Germany.

Jana et al. [77] developed a redox nanozyme (CMOR@4T1) for synergistic PTT and immunotherapy. As depicted in Figure 14, a Cu-doped MoO_x_ (CMO) nanozyme was initially synthesized using a metal–polyphenol coordination method. Subsequently, it was coated with the 4T1 cell membrane and loaded with R848, resulting in CMO-R@4T1. Following the injection of different NPs into tumor-bearing mice at the 12 h mark, the CMO-R@4T1 and CMO groups exhibited the highest fluorescence intensity, respectively, with the signal of CMO@4T1 being approximately 1.6 times higher. This suggests that CCm enhance the NPs’ targeting ability, allowing for their effective accumulation at tumor sites. In a bilateral tumor mouse model, treatment with CMO-R@4T1 combined with NIR-II irradiation significantly inhibited tumor growth and enhanced CD4^+^ and CD8^+^ T cell infiltration in both primary and distant tumors.

### 4.6. Hybrid Membrane-Coated Nanoparticles (Hym@NPs)

By fusing cell membranes isolated from various cell sources into a hybrid membrane (Hym), it is possible to integrate multifunctionality into a single platform. Consequently, creating Hym@NPs opens up broad prospects for the application of bionic NPs in tumor therapy. Table 7 presents a compilation of Hym@NPs utilized in cancer therapy.

Ye et al. [85] developed PNMAuDIs to enhance the chemo-PTT treatment efficacy in breast cancer therapy. In their research, Hym (named PNM) was synthesized from PLTm and Nm through 10 min of sonication, with the resulting PNM subsequently enveloping a gold nanocage core loaded with DOX and ICG. Further analysis revealed that this fusion retained cell membrane proteins from both PLTs and neutrophils. Consequently, the PNMAuDIs demonstrated a strong affinity towards CTCs and migrating tumor-derived exosomes, markedly reducing tumor growth and metastasis. Similarly, Chen and colleagues [138] crafted a Hym@NP combining RBCm and CCm, named Hyb-NP. This construct was designed to extend circulation time and enhance tumor recognition, encapsulating monensin—a metastasis inhibitor. Hyb-NP efficiently delivers monensin to the Golgi apparatus, thereby extending circulation time and significantly reducing spontaneous metastasis in an orthotopic breast cancer model.

**Figure 15:**
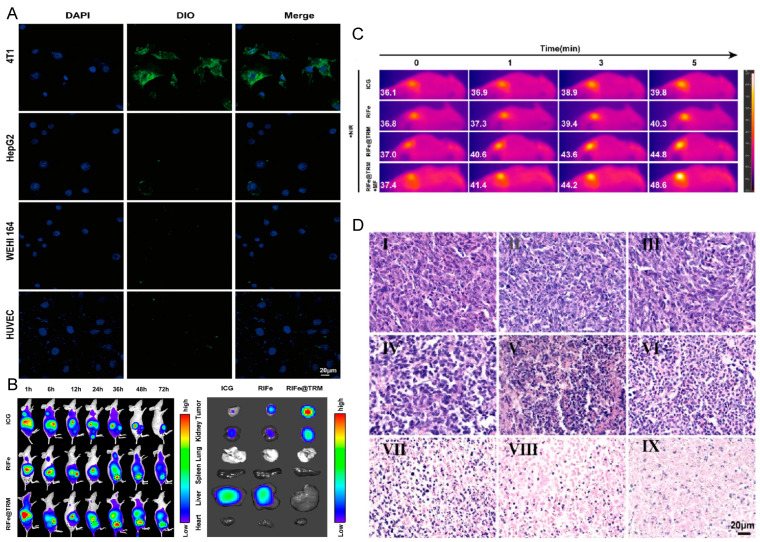
(**A**) In vivo real-time fluorescence images (Scale bar: 20 μm). (**B**) In vitro fluorescence images of tumors and major organs. (**C**) Infrared thermography of tumor-bearing mice. (**D**) H&E staining of the tumor sections (I: Saline, II: Fe_3_O_4_, III: R837, IV: ICG, V: RFe, VI: RIFe + NIR, VII: RIFe@TRM, VIII: RIFe@TRM + NIR, IX: RIFe@TRM + NIR + MF, Scale bar: 20 μm). Reprinted with permission from Ref. [86]. Copyright © 2024 American Chemical Society: Washington, DC, USA.

Similarly, Zhang et al. [86] focused on developing TRM (CCm-Mm) hybrid Fe_3_O_4_ NPs loaded with ICG and imiquimod (R837), named RIFe@TRM, for a combination therapy approach in breast cancer treatment. In vitro studies demonstrated that the TRM hybrid membrane coating exhibited excellent self-targeting capabilities, as evidenced by enhanced fluorescence in 4T1 cells indicative of a higher uptake, compared to others, when incubated with DiO-labeled RIFe@TRM (Figure 15). These fluorescence images further suggest that TRM significantly extends the circulation life of the NPs. Upon administering RIFe@TRM combined with laser irradiation and a magnetic field (MF) for 5 min in a mouse model, the temperature in the targeted area increased to 48.6 °C, leading to pronounced tumor cell apoptosis and necrosis, as confirmed by H&E staining.

**Figure 16:**
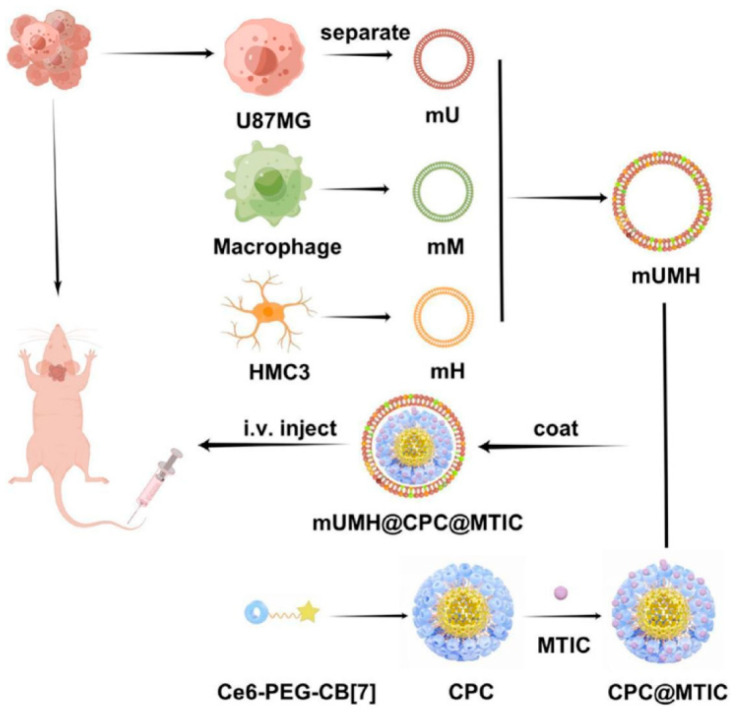
Design of tumor microenvironment targeting for glioblastoma multiforme treatment via hybrid cell membrane coating supramolecular micelles. Reprinted with permission from Ref. [90]. Copyright © 2024 Elsevier B.V: Amsterdam, The Netherlands.

Recently, Huang et al. [90] innovatively developed triple-fused cell membranes (mUMH) that encapsulate supramolecular micelles, enabling them to cross the BBB for GBM treatment (Figure 16). In their research, cell membranes from glioblastoma, macrophages, and microglia (mH) were selected and fused in a 2:1:1 ratio through co-extrusion, aiming to boost brain tumor targeting efficiency. The mUMH demonstrated superior intracellular uptake and tumor targeting capabilities compared to individual membrane types.

A wide range of cell membrane combinations has been investigated for preparing Hym to encapsulate nanomaterials. Beyond the examples previously discussed, research has explored hybrids of cancer–cancer [91], DC–cancer [92], RBC–PLT [139], mitochondria–cancer [140], bacteria–cancer [141], and embryonic–bacteria [142], among others.

## 5. Advantages and Limitations

**Figure 17:**
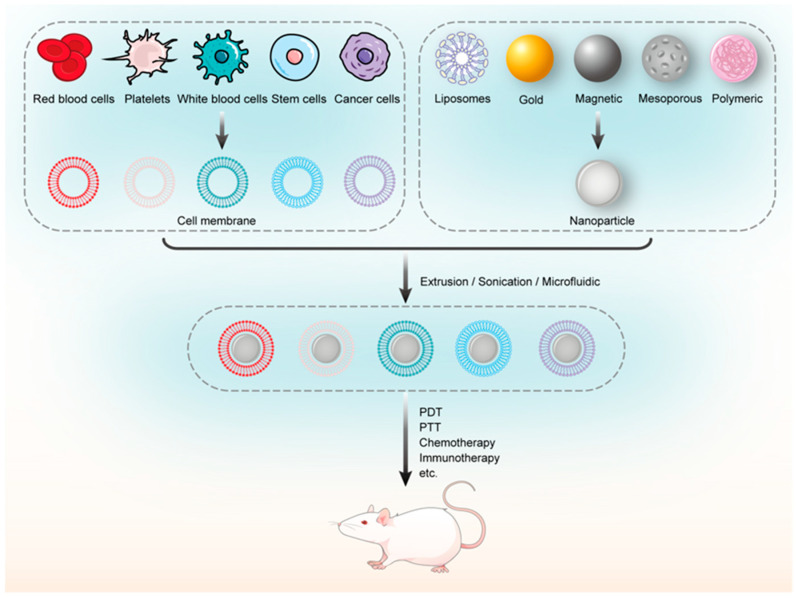
Different classification of CMC@NPs used for cancer therapy (Created by authors using Adobe Illustrator 2023^®^ software).

Biomimetic nanoplatforms coated with cell membranes possess tremendous potential as delivery nanocarriers, fusing various cell types with versatile nanomaterials and encapsulating diverse drugs (Figure 17). The cell membrane bestows biomimetic properties and unique functions upon conventional NPs. The use of CMC@NPs in cancer therapy has yielded encouraging achievements, offering benefits such as immune evasion, tumor targeting, and enhanced immune responses.

Most NPs, as exogenous substances, are quickly recognized and cleared by the immune system upon entry into the body. The application of a membrane coating effectively disguises NPs as “self” entities, enabling them to avoid detection by the reticuloendothelial system and extending their circulation time in the bloodstream. This results in enhanced extravasation into tumor tissue through the Enhanced Permeability and Retention (EPR) effect. Several types of CMC@NPs have been shown to increase biocompatibility and evade immune detection, notably RBCm@NPs. An experiment by Su et al. [143] demonstrated that RBCm@NPs extended circulation nearly 5.8 times longer than bare NPs.

Tumor targeting is essential for a drug delivery system to achieve maximum efficacy in cancer treatment. Scientists are increasingly focusing on enhancing active targeting modification, as opposed to passive targeting, which may lead to drug resistance. Certain cell types, such as cancer cells, WBCs, and PLTs, innately possess tumor homing abilities. This phenomenon can be attributed to proteins or ligands on the cell membrane that recognize and bind to tumor cell receptors, facilitated by recruitment through chemoattractants. Consequently, the cell membrane has been extensively exploited for active targeting modification. Notably, CCm@NPs, whether used solely or in combination with other membranes, demonstrate improved tumor-specific targeting capabilities and an enhanced cellular uptake. Han et al. [144] developed a glioma membrane-coated PEI nano-core loaded with plasmid DNA, indicating high accumulation and transfection efficiency in tumor cells, leading to a significant reduction in tumor size. NPs coated with cell membranes enhance tumor targeting efficiency, offering the potential to achieve precision medicine and minimize side effects.

Coating NPs with WBCm or CCm holds promise for immune activation. DCm@NPs can mimic APCs and promote the differentiation of CD4^+^/CD8^+^ T cells; Tm@NPs can bind to immunosuppressive molecules (e.g., TGF-β) and PD-L1 on tumor cells, effectively restoring T cells’ cancer-killing capabilities. Similarly, NPs coated with TAMm can drive M1 phenotype polarization by depleting macrophage colony-stimulating factors (e.g., CSF1) secreted by tumor cells, thereby reshaping the TME. Furthermore, following lysosomal degradation or laser irradiation, CCm@NPs discharge CCm fragments that serve as tumor antigens, encouraging the maturation of APCs and stimulating the immune response.

Cell membrane-coated biomimetic NPs have shown exceptional therapeutic efficacy in various anti-tumor strategies, owing to their unique advantages. However, limitations exist as well. Specifically, RBCm@NPs offer high biocompatibility and an extended plasma half-life, yet their tumor-targeting capability is limited. PLTm@NPs can target tumors effectively and sustain circulation, but carry a potential risk of thrombosis. WBCm@NPs demonstrate precise targeting and immune activation, but exhibit high heterogeneity. SCm@NPs possess a strong affinity for tumors, yet lack the desired specificity. CCm@NPs are capable of actively targeting cancer cells and modulating the immune response, but raise safety concerns, such as oncogenic risks. Hym@NPs combine various functionalities, yet may lead to diminished effects.

During the manufacture of CMC@NPs, the scarcity of certain source cells can lead to increased costs for membrane harvesting. Moreover, the thorough removal of intracellular contents and genetic material is essential to ensure membrane purity, especially with cancer cells, due to potential carcinogenic risks. Furthermore, keeping the preparation process at a low temperature is vital to maintain the stability and integrity of surface proteins, which are crucial for biological functions and minimizing endogenous immune reactions. Although various methods for preparing CMC@NPs exist, most are confined to lab-scale experiments, posing challenges for upscaling to industrial production because of their laboriousness, low efficiency, and inconsistent results. To fulfill clinical application standards, the continuous improvement and optimization of the preparation process is necessary.

The storage of CMC@NPs presents another challenge for clinical applications. Strategies are needed to ensure their long-term preservation and to prevent a loss of stability and therapeutic effectiveness. Furthermore, rigorous protocols are required to protect CMC@NPs from contaminants such as endotoxins, viruses, and pyrogens.

Although CMC@NPs have shown exhilarating potential in laboratory settings with model animals, the transition to effective therapeutic applications in human patients is fraught with significant challenges, especially in terms of operational complexity, time efficiency, and financial cost. The process spanning from the extraction of cell membranes to the preparation of drug-encapsulated NPs necessitates meticulous operations, demanding high technical proficiency, precise manipulation, and stringent quality control, increasing both complexity and technical demands in clinical settings. Consequently, the procedures must be streamlined and standardized. Furthermore, given the urgency of treatment for cancer patients, the time efficiency in preparing CMC@NPs is critical, as any delays could significantly impact therapeutic outcomes. Scientists are investigating pre-preparation and storage techniques for cell membrane biomimetic NPs or contemplating the use of generic cell membranes suitable for multiple patients. However, these strategies also face challenges related to long-term storage stability and the uncertainty of treatment outcomes due to individual patient differences. Interim therapeutic measures may be necessary to compensate for the preparation time. Finally, the substantial economic cost associated with this technology—stemming from the requirement for specialized equipment, materials, and skilled labor—considerably limits its accessibility, which further complicates the process of translating these advancements into clinical practice.

## 6. Conclusions and Prospects

This review aims to outline the preparation, features, functions, and potential applications of CMC@NPs, and several challenges were discussed. With the efforts of scientists, cell membrane-based DDSs have made significant progress in recent years. As innovative drug carriers, cell membrane-camouflaged NPs have demonstrated tremendous potential in cancer therapy, offering advantages such as excellent biocompatibility, immune evasion, prolonged blood circulation, a high biodistribution profile, enhanced immune response, active tumor targeting, and increased drug accumulation at tumor sites.

Although the transition of CMC@NPs from laboratory research to translational medicine faces numerous challenges, there is an undeniable optimism that advances in cell engineering and biomimetic nanotechnology will gradually address these issues. Recently, scientists have discovered a method to improve the full coating ratio of CMC@NPs by enhancing membrane fluidity [145]. In another study, bacteria were co-cultivated with host cells and subsequently exposed to UV light, triggering apoptosis and resulting in the spontaneous coating of bacteria with cell membranes [146]. Both studies pave the way for more efficient and easily adaptable coating techniques.

Personalized healthcare represents a promising direction for the clinical application of CMC@NPs. NPs coated with self-derived cell membranes can maximally avoid immune system activation. The development of Hym@NPs and advances in biological membrane modification offer boundless opportunities for this innovative biomimetic platform. Moreover, NPs based on EVs, such as exosomes and microvesicles, are attracting attention for their cargo transport capabilities and inherent functional properties.

In summary, although there are still some hurdles to be solved from bench to clinical practice, cell membrane-based biomimetic systems represent a feasible approach to decorate nanoparticles and hold great promise for cancer therapy.

## Figures and Tables

**Table 1:** List of recent studies on CMC@NPs across different cancer models.

Type of CMC@NPs	Cancer Models	References
RBCm@NPs	Melanoma	[30,31]
	Cervical cancer	[32,33]
	Breast cancer	[34,35]
	Colon cancer	[23]
	Liver cancer	[36,37]
PLTm@NPs	Melanoma	[38,39]
PLTm@NPs	Breast cancer	[40,41,42]
	Colon cancer	[42,43]
	Liver cancer	[44]
Nm@NPs	Breast cancer	[7,28]
	Liver cancer	[45]
	Pancreatic cancer	[46,47]
Mm@NPs	Breast cancer	[48,49,50]
	Osteosarcoma	[51]
	Glioma	[52]
Tm@NPs	Melanoma	[53,54]
	Liver cancer	[55]
	Glioma	[56]
	Lymphoma	[57]
DCm@NPs	Melanoma	[58,59]
	Breast cancer	[60,61]
	Liver cancer	[62]
	Glioma	[63]
NKm@NPs	Breast cancer	[64]
	Liver cancer	[65]
	Glioma	[66,67]
SCm@NPs	Liver cancer	[68]
	Bone cancer	[69]
	Prostate cancer	[70]
CCm@NPs	Melanoma	[71,72,73]
	Cervical cancer	[74,75]
	Breast cancer	[76,77]
	Colon cancer	[78]
	Osteosarcoma	[79,80]
	Colorectal cancer	[81]
	Oral squamous cell carcinoma	[82]
	Bladder cancer	[83]
Hym@NPs	Melanoma	[84]
	Breast cancer	[85,86,87]
	Liver cancer	[88]
	Colon cancer	[89]
	Osteosarcoma	[25]
	Glioma	[90,91,92,93]
	Ovarian cancer	[94]
	Gastric cancer	[95]

**Table 2:** List of RBCm@NPs applied in cancer therapy.

Source Cell	Inner Cores	Therapeutics	Key Functions	References
RBCs	Curdlan	Chemotherapy, immunotherapy	Long circulation, immune evasion	[31]
	Melanin	PTT	Long circulation, good biocompatibility	[36]
	PLGA/AIEgen/Poly(I:C)	PDT, immunotherapy	Long circulation, homing to spleen	[30]
	Fe_3_O_4_@Cu_2−x_S	MRI, PTT	Immune evasion	[32]
	UCNP	Tumor imaging	Good biocompatibility	[34]
	Ag_2_S	MRI, SDT	Long circulation, good biocompatibility	[23]
	PLGA	Chemotherapy	Long circulation, immune evasion	[35]
	PEG-b-PDLLA	Chemotherapy, PDT	Long circulation	[33]

**Table 3:** List of PLTm@NPs applied in cancer therapy.

Source Cell	Inner Cores	Therapeutics	Key Functions	References
PLTs	PLA	Immunotherapy	Tumor targeting, good biocompatibility	[42]
	-	Chemotherapy, immunotherapy	Tumor targeting, responds to coagulation signals	[43]
	PLGA	PDT	Long circulation, tumor targeting	[40]
	Cu_2_O	PDT, cuproptosis	Long circulation, tumor targeting	[41]
	PLGA-ss-HA	Chemotherapy	Tumor targeting	[38]
	MSN	Chemotherapy	Tumor and damage vessel targeting	[44]
	MOF	Gene therapy	Tumor targeting	[102]
	MSN	Hypoxia-sensitive chemotherapy	Tumor and damage vessel targeting	[103]
	HGN	PTT	Long circulation, tumor targeting	[104]

**Table 4:** List of WBCm@NPs applied in cancer therapy.

Source Cell	Inner Cores	Therapeutics	Key Functions	References
Neutrophils	PPDG	Chemotherapy, immunotherapy	Inflammation targeting, immune induction	[7]
	IMN	Isolation and analysis of CTCs	Tumor targeting, immune evasion	[28]
	Lip-GEM	Nanosecond pulsed electric field, chemotherapy	Tumor targeting	[46]
	PLGA	PDT	Tumor targeting, immune evasion	[45]
	PEG-PLGA	Chemotherapy	Tumor targeting	[47]
Macrophages	PEG-PDPA	Immunotherapy	Tumor targeting, immune induction	[48]
	-	Immunotherapy	Tumor and macrophage targeting	[51]
	PLGA	Immunotherapy	Tumor targeting, BBB crossing, immune induction	[107]
	Fe_3_O_4_	Chemotherapy, phototherapy	Tumor targeting, immune induction	[49]
	Polydopamine	PTT, immunotherapy	Tumor and inflammation targeting	[108]
	PLGA	MRI, chemotherapy, chemodynamic therapy	Long circulation, BBB crossing, tumor targeting	[52]
	UCNP	PDT, immunotherapy	Tumor targeting, immune evasion, immune induction	[50]
T cells	AIE	Gene editing, PTT	Tumor targeting, BBB crossing, good biocompatibility	[56]
	HA	Chemotherapy, immunotherapy	Tumor targeting, immune induction and evasion	[53]
	MSN	Gene editing, PTT	Tumor targeting	[55]
	PLGA	Immunotherapy	Tumor targeting, immune induction	[54]
	PLGA	PTT	Tumor targeting	[57]
Dendritic cells	PLGA	Immunotherapy	Tumor targeting, BBB crossing, immune induction	[63]
	MSN	PDT, immunotherapy	Tumor targeting, immune induction	[62]
	PLGA	Immunotherapy	Immune induction	[58]
	AIE	PDT, immunotherapy	Tumor targeting, immune induction	[60]
	PLGA	Click chemistry, immunotherapy	Immune induction	[59]
	Polymer NPs	Immunotherapy, PTT	Immune induction and evasion	[61]
Natural killer cells	PLGA	Chemotherapy, immunotherapy	Long circulation, tumor targeting, BBB crossing	[66]
	AsHMS-TA/Fe^III^	Thermodynamic–chemodynamic therapy	Long circulation, tumor targeting	[65]
	AIE	Fluorescence imaging, PTT	Tumor targeting, BBB crossing	[67]
	PLGA	Fluorescence imaging, MRI	Tumor targeting	[64]
	mPEG-PLGA	PDT, immunotherapy	Tumor targeting, immune induction	[109]

**Table 6:** List of CCm@NPs applied in cancer therapy.

Source Cell	Inner Cores	Therapeutics	Key Functions	References
Cancer cells	Nanogel	Chemotherapy, immunotherapy	Homologous targeting, long circulation	[8]
	Silica	Chemotherapy	Homologous targeting	[81]
	Cu-ZnO	NO therapy	Tumor targeting, immune evasion	[74]
	MSN	Tumor vaccine, immunotherapy	Immune induction	[71]
	PEI	Tumor vaccine, gene editing	Homologous targeting, immune induction	[72]
	HMnO_2_	Immunotherapy, chemodynamic therapy	Long circulation, homologous targeting	[79]
	Fe-PDAP	SDT, immunotherapy	Tumor targeting	[76]
	CMO_x_	PTT, immunotherapy	Tumor targeting, immune evasion	[77]
	TiO_2_	SDT, PTT	Tumor targeting	[75]

**Table 7:** List of Hym@NPs applied in cancer therapy.

Source Cell	Inner Cores	Therapeutics	Key Functions	References
PLTm-Nm	Gold nanocage	Chemotherapy, PTT	High affinity for CTCs and exosomes	[85]
Mm-CCm	Fe_3_O_4_	Chemodynamic therapy, immunotherapy, PDT	Immune evasion, homologous targeting	[86]
Mm-CCm-mH	CPC@MTIC	Chemotherapy	BBB crossing, homologous and inflammation targeting	[90]
CCm-CCm	OA-LnNPs	PTT	BBB crossing, homologous targeting	[91]
DCm-CCm	HPMC	Chemotherapy, immunotherapy	Homologous targeting, immune induction	[92]
RBCm-CCm	TK-PPE	PTT, immunotherapy	Long circulation, homologous targeting	[94]
RBCm-CCm	PLGA	Chemotherapy, PDT	Long circulation, homologous targeting	[89]
WBCm-CCm	Fe_3_O_4_	Isolation and analysis of CTCs	Tumor targeting, immune evasion	[87]
Mm-Nm	Ag_2_S	Immunotherapy	Homologous and inflammation targeting, BBB crossing	[93]

## Abbreviations

NPs	Nanoparticles
DDSs	Drug delivery systems
PEGs	Glycol polymers
PLGA	Poly(lactic-co-glycolic acid)
CMC@NPs	Cell membrane-coated NPs
MRI	Magnetic resonance imaging
SDT	Sonodynamic therapy
RBCs	Red blood cells
RBCm	Red blood cell membrane
DOX	Doxorubicin
PDT	Photodynamic therapy
PTT	Photothermal therapy
HSCs	Hematopoietic stem cells
Nm	Neutrophil membrane
PLTs	Platelets
PLTm	Platelet membrane
R848	Resiquimod
TLR	Toll-like receptor
WBCs	White blood cells
WBCm	White blood cell membrane
DCs	Dendritic cells
APCs	Antigen presenting cells
DCm	Dendritic cell membrane
BBB	Blood–brain barrier
NKs	Natural killer cells
SCs	Stem cells
CTCs	Circulating tumor cells
Mm	Macrophage membrane
TME	Tumor microenvironment
TAMs	Tumor-associated macrophages
H_2_O_2_	Hydrogen peroxide
CSF1	Colony-stimulating factor 1
CTLs	Cytotoxic T lymphocytes
ICG	Indocyanine green
CAR-T	Chimeric antigen receptor T
HCCs	Hepatocellular carcinomas
AIE	Aggregation-induced emission
MHCs	Major histocompatibility complexes
MSCs	Mesenchymal stem cells
ROS	Reactive oxygen species
ICD	Immunogenic cell death
Hym	Hybrid membrane
Nm@NPs	Neutrophil membrane-coated nanoparticles
CCm@NPs	Cancer cell membrane-coated nanoparticles
RBCm@NPs	Red blood cell membrane-coated nanoparticles
PLTm@NPs	Platelet membrane-coated nanoparticles
WBCm@NPS	White blood cell membrane-coated nanoparticles
MSCm@NPs	MSC membrane-coated nanoparticles
SCm@NPs	Stem cell membrane-coated nanoparticles
Hym@NPs	Hybrid membrane-coated nanoparticles
Mm@NPs	Macrophage membrane-coated nanoparticles
Tm@NPs	T cell membrane-coated nanoparticles
DCm@NPs	Dendritic cell membrane-coated nanoparticles
NKm@NPs	Natural killer cell membrane-coated nanoparticles
